# Efficacy and safety of fixed dose combination of arterolane maleate and piperaquine phosphate dispersible tablets in paediatric patients with acute uncomplicated *Plasmodium falciparum* malaria: a phase II, multicentric, open-label study

**DOI:** 10.1186/s12936-015-0982-y

**Published:** 2015-11-25

**Authors:** Offianan Andre Toure, Stephen Rulisa, Anupkumar R. Anvikar, Ballamudi S. Rao, Pitabas Mishra, Rajinder K. Jalali, Sudershan Arora, Arjun Roy, Nilanjan Saha, Sunil S. Iyer, Pradeep Sharma, Neena Valecha

**Affiliations:** Malariology Department, Institut Pasteur, Abidjan, Côte d’Ivoire; Department of Clinical Research, School of Medicine, Kigali University Teaching Hospital, University of Rwanda, Butare, Rwanda; National Institute of Malaria Research, New Delhi, India; Department of Medicine, Tata Main Hospital, Jamshedpur, Jharkhand India; Department of Paediatrics, Ispat General Hospital, Rourkela, Odisha India; Medical Affairs, Clinical Research and Global Head Pharmacovigilance, Ranbaxy Laboratories Ltd, Gurgaon, Haryana India; Corporate Office, Ranbaxy Laboratories Ltd, Gurgaon, Haryana India; CDM and Biostatistics, Ranbaxy Laboratories Ltd, Gurgaon, Haryana India; Medical Global Marketing Corporate Office, Ranbaxy Laboratories Ltd, Gurgaon, Haryana India; Clinical Pharmacology and Pharmacokinetics, Ranbaxy Laboratories Ltd, Gurgaon, Haryana India

**Keywords:** Artemisinin combination therapy, Arterolane maleate, Piperaquine phosphate, Malaria, Paediatric, PCT, FCT, PCR-corrected ACPR, Fixed dose combination

## Abstract

**Background:**

The World Health Organization (WHO) recommends artemisinin combination therapy (ACT) for the treatment of uncomplicated *Plasmodium falciparum* malaria. The present study investigated the efficacy and safety of fixed dose combination (FDC) of arterolane maleate 37.5 mg and piperaquine phosphate (PQP) 187.5 mg dispersible tablets in paediatric patients aged 6 months to 12 years.

**Methods:**

Male and female patients aged 6 months to 12 years who were confirmed cases of *P. falciparum* mono-infection with fever or documented history of fever in the previous 24 h were included. The patients were administered FDC of arterolane maleate and PQP as single daily doses for three consecutive days based on their age. The primary efficacy outcome was proportion of patients with polymerase chain reaction (PCR)-corrected adequate clinical and parasitological response (ACPR) on day 28. Safety was analysed based on adverse events (AE), laboratory abnormalities and abnormalities on electrocardiograph.

**Results:**

A total of 141 eligible paediatric patients received FDC of arterolane maleate and PQP in a 42-day follow-up study. All the enrolled patients (141) were included in intention to treat (ITT) and safety analyses, and 126 patients were considered in per protocol (PP) population. The PCR-corrected ACPR on day 28 was achieved in all patients (100 %; 95 % CI 97.11–100) included in PP population. The median parasite clearance time (PCT) and fever clearance time (FCT) were 24 h (95 % CI 18.0–24.0) and 10 h (95 % CI 4.0–18.0), respectively. The most frequently reported clinical AE was vomiting. Majority of the AEs were mild to moderate in severity and resolved without sequelae. No patient was discontinued for any QTc (corrected QT interval) prolongation. No deaths or serious AEs were reported during the study.

**Conclusion:**

The findings from this study showed that FDC of arterolane maleate and PQP effectively cures *P. falciparum* malaria and attains acceptable level of cure by day 28 in paediatric patients. The efficacy and safety results observed in children warrants further studies on FDC of arterolane maleate and PQP dispersible tablets.

Trial Registration: Clinical Trial Registry India: CTRI/2009/091/000531

## Background

According to the World Health Organization (WHO) estimates, released in December 2014, there were about 198 million cases of malaria in 2013 and an estimated 584,000 deaths. The burden is heaviest in the WHO African Region, where an estimated 90 % of all malaria deaths occur, and in children under 5 years of age, who account for 78 % of all deaths [1]. Early effective treatment of malaria is the cornerstone of malaria control. *Plasmodium* resistance to anti-malarial medicines is one of the major obstacles in the fight against malaria. Artemisinin combination therapy (ACT) is the WHO-recommended first-line treatment for uncomplicated falciparum malaria in all endemic regions [2, 3].

The clinical effectiveness of the artemisinin derivatives in ACT is due to rapid clearance of parasitaemia and rapid resolution of symptoms, by reducing parasite numbers. However, artemisinin derivatives are derived from plant source, so harvesting and extraction costs remain variable. This leads to fluctuation in the supply of artemisinins [2, 5, 6]. Therefore, there exists an urgent need for development of novel, preferably fully synthetic anti-malarial drugs. Arterolane maleate is a new synthetic trioxolane that is easy to synthesize, inexpensive, and rapidly acting oral anti-malarial drug. In ACT, artemisinin derivatives are used in combination with a long acting, more slowly eliminated partner drug that prevents recrudescent infections. Piperaquine phosphate is a proven effective and well-tolerated anti-malarial drug. The tolerability, efficacy and pharmacokinetic profile and low cost of piperaquine phosphate make it a promising partner drug for use as part of ACT [4, 7, 8]. Arterolane maleate has been combined with piperaquine phosphate (long-acting anti-malarial) in a fixed dose combination.

Efficacy, safety, tolerability, and pharmacokinetics of arterolane maleate and piperaquine phosphate co-administration were established in Phase I [9] and II studies. A phase II study assessed the anti-malarial efficacy and safety of a combination of 150 mg of arterolane maleate and 750 mg of piperaquine phosphate in comparison to Coartem^®^ (artemether-lumefantrine, AL) in patients with acute uncomplicated falciparum malaria. In this open-label, randomized, multicentric, parallel group, clinical trial, 240 adult patients were randomized to receive arterolane maleate and piperaquine phosphate (160 patients) or AL (80 patients). No treatment failure was noted in the arterolane maleate and piperaquine phosphate group, while one patient receiving AL was treatment failure on day 28. There was no difference in the median parasite clearance time (30 h in both groups) or median fever clearance time (24 h in both groups) after administration of the two study treatments [10]. Subsequently, fixed dose combination (FDC) of arterolane maleate 150 mg + piperaquine phosphate 750 mg tablet was developed. Two phase III studies with FDC of arterolane maleate 150 mg + piperaquine phosphate 750 mg tablet have been conducted: one in adults and adolescent patients with falciparum malaria and another in adult patients with *Plasmodiumvivax* infection. Based on efficacy and safety results in these studies, FDC of arterolane maleate 150 mg + piperaquine phosphate 750 mg tablet has been approved in India for the treatment of falciparum and vivax malaria in adult patients.

Administration of ACT to infants and small children can be difficult and time consuming. Specially formulating anti-malarials for this vulnerable population is vital to ease administration and help ensure that an accurate dose is received. Easing administration may enhance adherence, improving therapeutic outcomes in infants and young children and preserving the efficacy of ACT [11]. According to the “Guidelines for the Treatment of Malaria, WHO 2010” the lack of paediatric drug formulations is a major impediment for the adequate treatment of young children as it necessitates the splitting of adult tablets leading to inaccurate dosing [2]. Realizing the need for appropriate paediatric formulation, a FDC of arterolane maleate 37.5 mg and piperaquine phosphate 187.5 mg dispersible tablet has been formulated.

A comparative bioavailability study of paediatric formulation, i.e., FDC of arterolane maleate 37.5 mg and piperaquine phosphate 187.5 mg dispersible tablet (four tablets) with co-administered conventional release arterolane maleate 150 mg (three tablets of 50 mg each) and piperaquine phosphate 750 mg was conducted in 48 healthy male subjects. Based on the pharmacokinetic results, oral bioavailability of FDC of arterolane maleate 37.5 mg and piperaquine phosphate 187.5 mg dispersible tablet was comparable to co-administered conventional release arterolane maleate 150 mg and piperaquine phosphate 750 mg in healthy, adult male subjects under fasting condition.

Data from phase III studies in adults and adolescents have demonstrated that the FDC of arterolane maleate 150 mg + piperaquine phosphate 750 mg tablet has a good safety profile. Because children are the population primarily affected by malaria, the present phase II study investigated the anti-malarial efficacy and safety of FDC of arterolane maleate 37.5 mg and piperaquine phosphate 187.5 mg dispersible tablets in paediatric patients with acute uncomplicated falciparum malaria.

## Methods

### Study site and enrolment

This phase II, multicentric, open-label study of 42 days’ duration was carried out between July 2010 to November 2012 at six sites across India and Africa. The patients were recruited from three hospitals each in India (Ispat General Hospital, Rourkela, Orissa; Tata Main Hospital, Jamshedpur, Jharkhand; and, Mahadevi Birla Hospital and Research Centre, Ranchi, Jharkhand) and Africa (General Hospital of Ayame, Ivory Coast; North Abobo General Hospital, Ivory Coast; and, Ruhuha Health Centre, Bugesera District, Rwanda).

### Inclusion and exclusion criteria

Children of either gender aged between 6 months and 12 years who presented with clinical symptoms of malaria were screened for study eligibility after assent was obtained from the patient (where possible) or informed consent from parents/guardians. Children with microscopically confirmed acute uncomplicated falciparum malaria, with parasite density ranging from 1000 to 100,000 asexual parasites/μL (both inclusive) and an axillary temperature ≥37.5 °C or history of fever in the past 24 h, were included in the study. Additional inclusion criteria were minimum body weight of 5 kg; ability to take drugs under study by the oral route, absence of severe malnutrition (defined as a child whose weight-for-height is below −3 standard deviation or less than 70 % of the median of the NCHS/WHO normalized reference values or who had symmetrical oedema involving at least the feet, minimum haemoglobin level ≥8 g/dL; and, willingness and ability to comply with the study protocol for the duration of the study; residence within a reasonable distance of the investigational site so that attendance of all study visits and follow-up by medical staff were logistically feasible.

Exclusion criteria included mixed *Plasmodium* infection; severe malaria; presence of general danger signs of severe malaria among children <5 years old (as per WHO); infants with a history of hyperbilirubinaemia during the neonatal period; pregnant and lactating females (between the age of 8 and 12 years); ongoing prophylaxis with drugs having anti-malarial activity, such as cotrimoxazole, for the prevention of *Pneumocystis carinii* pneumonia in children born to HIV + women; any anti-malarial treatment during 1 month prior to screening; use of concomitant medications that could induce haemolysis or haemolytic anaemia from the WHO list of essential drugs; known allergy to artesunate, artemether, artemisinin-derived products, piperaquine phosphate or any other related drug; and, participation in any investigational drug study during 30 days prior to screening. Patients with electrocardiogram (ECG) abnormalities with clinical significance or relevance that required urgent management were excluded from the study. These abnormalities included QTc interval >450 ms at screening and cardiac conduction disorders, with the exception of right bundle branch block. Patients with known significant renal or hepatic impairment indicated by the following laboratory evaluations at screening: serum creatinine >1.5 × upper limit of normal (ULN), aspartate transaminase >2.5 × ULN, alanine transaminase >2.5 × ULN and serum bilirubin >3 mg/dL; patients who have had a splenectomy; known history of HIV infection or other immunosuppressive disorders; evidence of clinically significant cardiovascular, pulmonary, metabolic, gastrointestinal, neurological, or endocrine diseases, malignancy, or other abnormalities; history of epilepsy/convulsions; evidence of gastrointestinal dysfunction that could alter absorption or motility (e.g., diarrhoea defined as >3 episodes of watery stools in the previous 24 h or patients who have had three episodes of vomiting within 24 h prior to screening); any other underlying disease that could compromise the diagnosis and the evaluation of the response to the study medication (including clinical symptoms of immunosuppression, tuberculosis, bacterial infection, cardiac or pulmonary disease) were also excluded from the study.

### Ethical considerations

The study was conducted as per the study protocol that was reviewed and approved by the Institutional Ethics Committees/Institutional Review Boards of the participating study sites and regulatory authorities. This clinical study was conducted in accordance with the Good Clinical Practice, applicable regulatory requirements, and Declaration of Helsinki. Written informed consent was obtained from all parents/guardians of the patients. Assent was acquired from children wherever it was applicable. This study is registered with Clinical Trial Registry India (CTRI/2009/091/000531).

### Study treatments

Patients were administered FDC of arterolane maleate 37.5 mg and piperaquine phosphate 187.5 mg dispersible tablets as single daily doses for three consecutive days based on their age (age group I, 6 months to <2 years: one tablet; age group II, 2 to <6 years: two tablets; and, age group III, 6 to ≤12 years: 3 tablets) by the qualified staff under supervision. Each tablet was dissolved in 10 mL of drinking water with continuous swirling. The study medication was administered irrespective of meals. If a patient vomited or regurgitated (for small children) within 1 h of receiving any dose of investigational product on any of the dosing days, the patient was administered a second full dose (repeat dose). If the patient vomited again within 30 min of receiving the repeat dose, the patient was withdrawn from the study and given rescue treatment. Re-dosing in case of vomiting was allowed only once during the study.

### Clinical and laboratory procedures

Initial screening was offered to patients who presented with fever or history of fever and eligible patients were hospitalized for a treatment period of 3 days (day 0, 1 and 2: day 0 being the first day of study medication dosing). Patients were discharged on day 3 and follow-up assessments were done on weekly intervals up to day 42.

Physical examination, vital signs, body temperature, and clinical assessment were done at screening, on therapy and at all follow-up visits. Body temperature was recorded at 6 h intervals (or adjusted to the closest possible 6 h interval to make the schedule consistent with routine care) following the first dose of study medication until temperature normalized and remained normal for 24 h, and at every visit thereafter. A 12-lead electrocardiograph was performed on screening day 0, 1, 2, and on any unscheduled day if patient reported with fever and at follow up visits if ECG was abnormal.

Laboratory evaluations (haematology, biochemistry and urinalysis) were performed during study and at follow-up visits. Clinically significant laboratory abnormalities on any visit were repeated during the next follow-up visit or earlier, if clinically indicated. Adverse events were reported for the time of study medication administration and at all follow-up visits.

### Parasitological assessments and parasite genotyping

Thick/thin blood smears were collected at the time of screening, pre-dose at day 0 and at 6 h intervals (or adjusted to the closest possible 6 h interval to make the schedule consistent with routine care) following first dose of administration until two consecutive negative smears were recorded, thereafter at day 3 and follow-up visits.

Filter paper samples for molecular marker studies [polymerase chain reaction (PCR) genotyping] were collected on screening and in the event of re-appearance of parasites, confirmed by microscopy, or on any other day if a patient returned with fever. PCR analysis was done at a central laboratory.

### Drug concentration measurement

Venous blood samples were collected from each enrolled patient for pharmacokinetic analysis of arterolane maleate and piperaquine phosphate. The compounds were measured by LC–MS/MS methods validated as per US FDA Guidance for Industry: bioanalytical method validation, May 2001 [12]. An analysis was performed in compliance with Good Laboratory Practice regulations.

### Outcomes

The study was conducted according to WHO guidelines [13]. The primary efficacy outcome was proportion of patients with PCR-corrected adequate clinical and parasitological response (ACPR) on day 28. PCR-corrected ACPR was defined as patients who had clearance of parasitaemia, irrespective of axillary temperature, without previously meeting any of the criteria of early treatment failure (ETF) or late clinical failure (LCF) or late parasitological failure (LPF). Secondary efficacy outcomes included parasite clearance time (PCT), defined as time in hours from the initiation of therapy until the first of two successive negative smears are obtained; fever clearance time (FCT) defined as the time from first dosing until temperature normalizes (<37.5 °C) and remains normal for 24 h, and at every follow up visit thereafter; proportion of patients with PCR-uncorrected ACPR on day 28; proportion of patients with PCR-corrected and PCR-uncorrected ACPR on day 42 and the number of gametocytes count. Safety endpoints were adverse events or clinically significant changes in laboratory parameters, physical examination, ECG or vital signs.

### Sample size determination

To have 100 evaluable patients in this study, 120 patients were planned to be enrolled, assuming an expected treatment failure rate of 4 % (supported by another in-house phase II study, cure rate of ~96 %), a 5 % level of significance and a precision of ±4 % [10]. This was done to ensure that the 95 % (exact binomial) confidence interval of cure rate would be between 90 and 99 %, well within the expected range. Additional 20 % patients were accounted for the dropouts during the study. However, while conducting the study in India, dropout rate of ~30 % was experienced on account of vomiting. Therefore, enrolment of additional patients was allowed at these sites to ensure availability of adequate evaluable data. Accordingly, a total of 141 patients were enrolled in the study.

### Statistical methods

The primary efficacy analysis was based on per-protocol population (PCR-corrected ACPR at day 28). The per-protocol population included all patients who completed a full course of study medication with a known efficacy endpoint and who did not violate the protocol in a way that might affect the efficacy analysis, i.e., the use of prohibited concomitant medication, the presence of significant disease or co-morbid illness, or major protocol violation. Intent-to-treat (ITT) and survival analyses were considered supportive. All the secondary endpoint (ACPR uncorrected at day 28, ACPR uncorrected/corrected at day 42, PCT, and FCT) analyses were done on ITT population, which included all patients who received at least one dose of study medication.

The proportion of patients having PCR-corrected ACPR at day 28 was estimated using 95 % exact (Clopper-Pearson) binomial confidence interval. The proportion of patients with PCR-uncorrected ACPR on day 28 and PCR-uncorrected ACPR at day 42 were analyzed similar to the primary endpoint. Time-to-event parameters (PCT and FCT) were assessed using survival analysis (Kaplan–Meier method). Patients with early withdrawal or those in whom parasite was not cleared or had fever at 72 h were censored at day 7 (168 h), i.e., next follow-up visit. These patients were considered failures in ITT analysis. Gametocyte counts at different visits were summarized using descriptive statistics, along with proportion of patients with zero gametocyte. Demographic and other baseline characteristics were summarized descriptively using mean, standard deviation, range, and count (%).

The safety population was defined as all patients who received any amount of study medication and had at least one assessment after dosing. The safety analyses included incidences of adverse events, summary statistics of laboratory data and ECG findings. All the analyses were performed using SAS software version 9.1.3 (SAS Institute, Cary, NC, USA) at 5 % two-sided level.

## Results

### Patient disposition, demographics and baseline characteristics

**Fig. 1:**
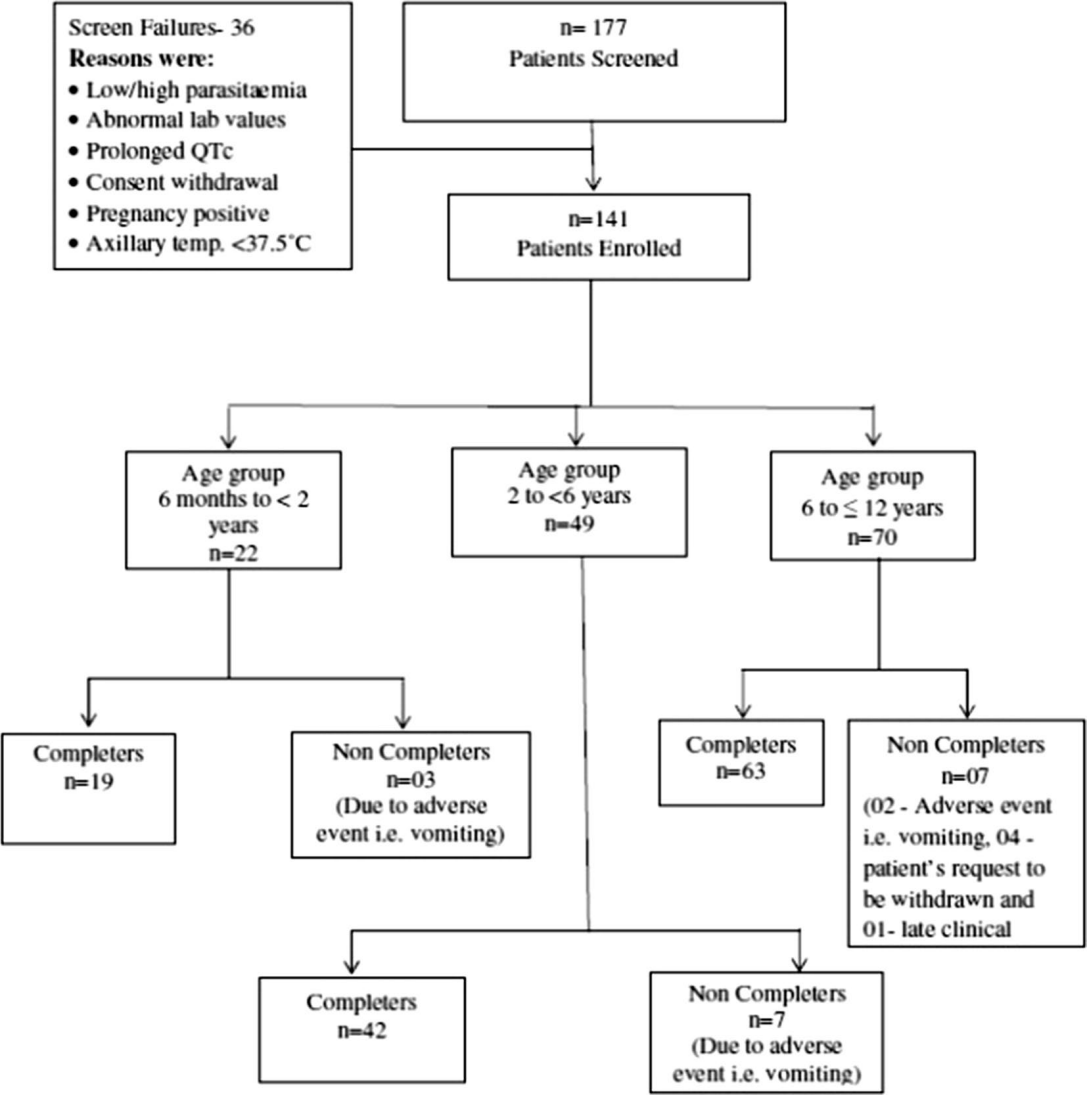
Patients’ disposition

A total of 141 patients were enrolled in the study to receive FDC of arterolane maleate and piperaquine phosphate dispersible tablets. Seventeen patients were withdrawn from study: 12 due to vomiting, four requested to be withdrawn and one was LCF at day 40; 124 patients completed day 42 of the study. However, 126 patients were considered in per-protocol population as the primary efficacy analysis was performed at day 28 (Fig. 1). The patient population had 73 (52 %) male and 68 (48 %) female patients. The mean age was 5.9 ± 3.45 years (Table 1).

**Table 1:** Demographic and baseline clinical characteristics

	FDC of arterolane maleate + piperaquine phosphate (n = 141)
Sex, n (%)	
Male	73 (52 %)
Female	68 (48 %)
Age (6 months to ≤12 years), n	141
Median	5.11
Mean ± SD (min, max)	5.9 ± 3.45 (0.06, 12.11)
Age group 1 (6 months to <2 years), n	22
Median	1.05
Mean ± SD (min, max)	0.9 ± 0.39 (0.06, 1.11)
Age group 2 (2 to <6 years), n	49
Median	4.00
Mean ± SD (min, max)	3.8 ± 1.07 (2.00, 5.11)
Age group 3 (6 to ≤12 years), n	70
Median	9.00
Mean ± SD (min, max)	8.8 ± 1.83 (6.00,12.11)
Race, n (%)	
African	123 (87 %)
Asian	18 (13 %)
Height (cm)	
Median	106.00
Mean ± SD (min, max)	105.7 ± 19.24 (58.00,146.00)
Weight (kg)	
Median	17.00
Mean ± SD (min, max)	18.5 ± 6.84 (7.00, 36.00)
Baseline axillary temperature (°C)	
Median	38.20
Mean ± SD (min, max)	38.4 ± 0.88 (36.50, 40.50)
No. of baseline *P. falciparum* asexual parasites (µL^−1^)	
Median	44,720.00
Mean ± SD (min, max)	45,428.6 ± 31,481.95 (1040.00, 99,860.00)

*SD* standard deviation, *Min* minimum, *Max* maximum

### Clinical and parasitological outcome

All the enrolled patients (141) were included in ITT and safety analyses. The primary efficacy analysis (PCR-corrected ACPR at day 28) was based on per-protocol population, comprised of 126 patients. All the secondary endpoint analyses were done on ITT population.

### Uncorrected and corrected ACPR on day 28

In per-protocol population, the PCR-corrected ACPR on day 28 was achieved in 126 patients (100 %; 95 % CI 97.11–100) (Table 2). The PCR-uncorrected ACPR remained same as corrected ACPR in this study since there was no treatment failure on or before day 28.

**Table 2:** Adequate clinical and parasitological response (ACPR) at day 28 (per-protocol population)

	n	Success, n (%)	95 % CI
Age group 1 (6 months to <2 years)	19	19 (100.0)	82.35, 100.0
Age group 2 (2 to <6 years)	42	42 (100.0)	91.59, 100.0
Age group 3 (6 to ≤12 years)	65	65 (100.0)	94.48, 100.0
Overall age (6 months to ≤12 years)	126	126 (100.0)	97.11, 100.0

**Fig. 2:**
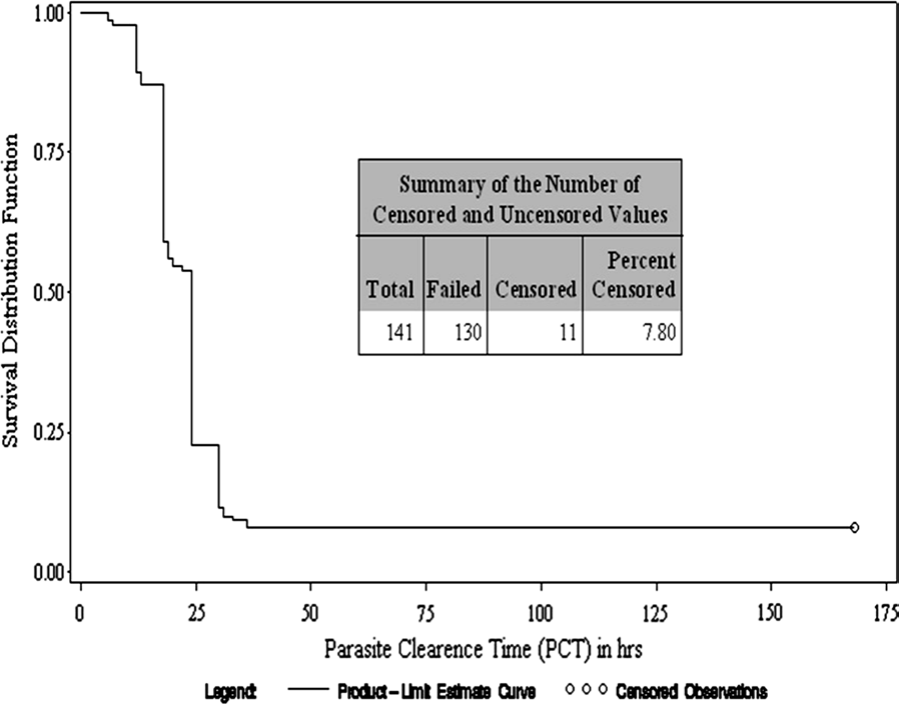
Survival analysis at day 42 (PCR-uncorrected) Kaplan–Meier method

Out of 141 patients, fifteen patients were withdrawn before day 28 and were considered failure. Therefore, the PCR corrected or uncorrected ACPR on day 28 in intent to treat population was achieved in 126 patients (89.36 %; 95 % CI 83.06–93.92). The Kaplan–Meier plot at day 28 supports 100 % PCR corrected or uncorrected response because all withdrawal cases were before day 28 (Fig. 2).

### Parasite clearance time (PCT) and fever clearance time (FCT)

**Fig. 3:**
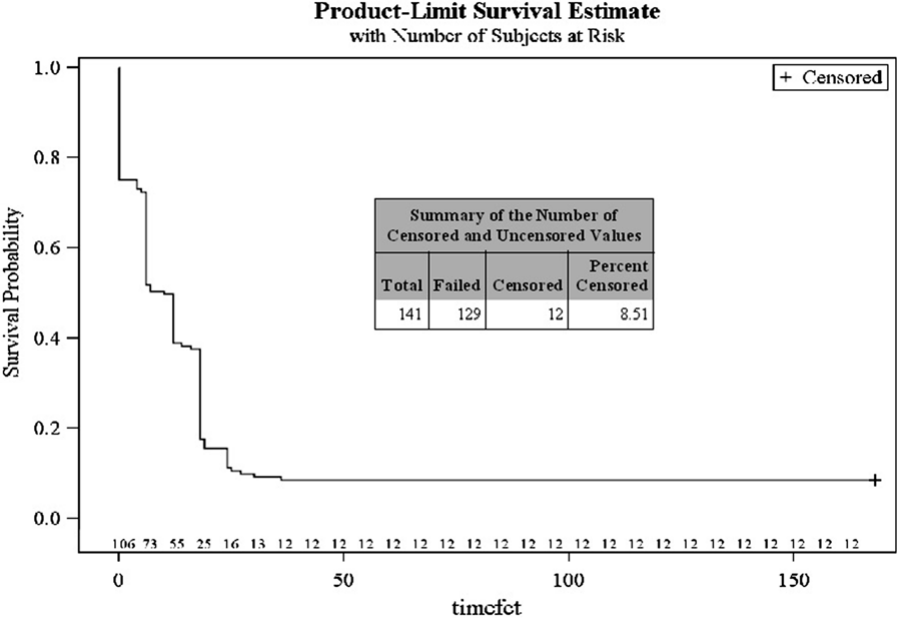
Time to parasite clearance (PCT)—Kaplan–Meier method

In the ITT population, the overall median PCT was estimated to be 24.0 h (95 % CI 18.0–24.0). The median PCT in patients of age group I (6 months to <2 years), age group II (2 to <6 years) and age group III (6 to ≤12 years) was estimated to be 22.0, 24.0 and 23.0 h, respectively (Fig. 3; Table 3).

**Table 3:** Parasite clearance time (PCT)

Statistical summary	Age group 1 (6 months to <2 years)	Age group 2 (2 to <6 years)	Age group 3 (6 to ≤12 years)
Censored	3 (13.6 %)	7 (14.3 %)	1 (1.4 %)
Summary (excluding censored values)			
n	19	42	69
Mean ± SD	21.5 ± 6.33	21.0 ± 6.81	21.2 ± 5.58
Min, max	12, 36	6, 31	12, 36
Time to parasite clearance (h)			
Quartile estimate (95 % confidence interval)			
25 %	18.0 (18.0, 20.0)	18.0 (13.0, 19.0)	18.0 (NE, NE)
50 % (median)	22.0 (18.0, 24.0)	24.0 (19.0, 24.0)	23.0 (18.0, 24.0)
75 %	30.0 (24.0, NE)	30.0 (24.0, 31.0)	24.0 (NE, NE)
Mean	23. 5	22.4	21.5

Overall the median PCT was estimated to be 24.0 h (18.0 to 24.0 h)

**Fig. 4:**
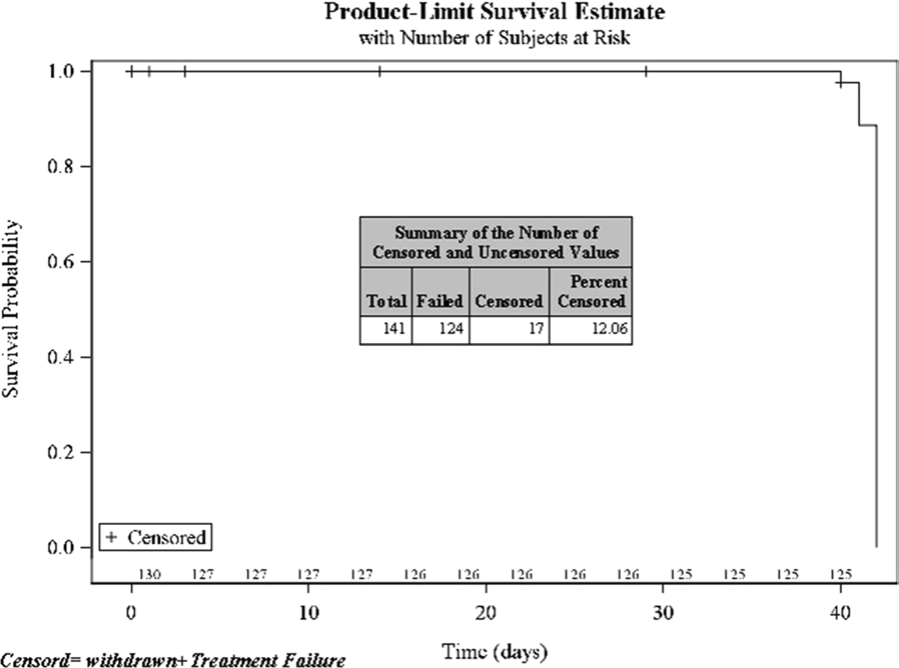
Time to fever clearance (FCT)—Kaplan–Meier method

Similarly, the overall median FCT was estimated to be 10.0 h (95 % CI 4.0–18.0 h). The median FCT in patients of age group I, II and III was estimated to be 12.0, 12.0 and 6.0 h, respectively (Fig. 4; Table 4).

**Table 4:** Fever clearance time (FCT)

Statistical summary	Age group 1 (6 months to <2 years)	Age group 2 (2 to <6 years)	Age group 3 (6 to ≤12 years)
Censored	3 (13.6 %)	6 (12.2 %)	3 (4.3 %)
Summary (excluding censored values)			
n	19	43	67
Mean ± SD	12.9 ± 9.37	9.1 ± 8.63	9.1 ± 7.86
Min, max	0, 36	0, 25	0, 30
Time to fever clearance (h)			
Quartile estimate (95 % confidence interval)			
25 %	6.0 (6.0, 12.0)	0.0 (0.0, 6.0)	4.0 (0.0, 6.0)
50 % (median)	12.0 (6.0, 19.0)	12.0 (6.0, 18.0)	6.0 (6.0, 12.0)
75 %	24.0 (12.0, NE)	18.0 (18.0, 24.0)	18.0 (12.0, 18.0)
Mean	16.0	11.0	10.0

Overall, median FCT was estimated to be 10.0 h (4.0–18.0 h)

*NE* not estimable

### Uncorrected and corrected ACPR on day 42

The uncorrected ACPR at day 42 was observed in 124 patients (87.9 %, 95 % CI 81.40–92.82) (Table 5). Among the 17 cases withdrawn from the study, there was one case of LCF at day 40 and in PCR testing it was noted as re-infection. Therefore, PCR-corrected ACPR at day 42 was 88.65 % (125/141) in ITT population at day 42. Similar result (88.73 %) was noted in survival analysis (Kaplan–Meier plot) (Fig. 2). PCR corrected or uncorrected ACPR at day 42 was 99.2 % (124/125; 95 % CI 95.62–99.98) in per protocol population at day 42,

**Table 5:** PCR uncorrected adequate clinical and parasitological response (ACPR) at day 42 (intention to treat population)

	Arterolane maleate and piperaquine phosphate	95 % CI
Overall Age (6 months to ≤12years) (n = 141)		
Success, n (%)	124 (87.9)	(81.40, 92.82)
Age group 1 (6 months to <2years) (n = 22)		
Success, n (%)	19 (86.4)	(65.09, 97.09)
Age group 2 (2 to <6years) (n = 49)		
Success, n (%)	42 (85.7)	(72.76, 94.06)
Age group 3 (6 to ≤12 years) (n = 70)		
Success, n (%)	63 (90.0)	(80.48, 95.88)

### Proportion of aparasitaemic patients on day 3 (PRR - Parasites Reduction Rate)

In ITT population, aparasitaemia was reported in 130 patients (92.20 %) within 72 h. Parasitological data were not available for 11 patients due to vomiting and were considered as failures.

### Gametocidal action

At baseline, gametocytes were reported in two out of 141 patients. After day 7, gametocytes were not detected in any of the patients.

### Pharmacokinetic outcome

**Fig. 5:**
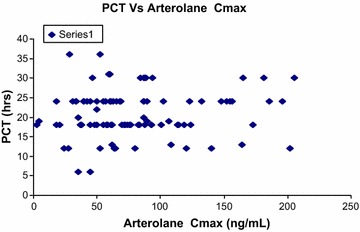
Parasite clearance time as a function of C_max_ of arterolane maleate

**Fig. 6:**
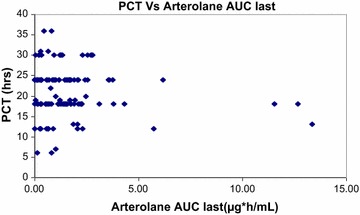
Parasite clearance time as a function of arterolane maleate exposure (AUC_last_)

Plasma concentration data were available from 130 out of 141 enrolled patients; 50 patients with intensive sampling and remaining 80 patients contributed to sparse sampling data. The pharmacokinetic parameters for arterolane maleate in sparse sampling could not be reliably calculated through non-compartmental analysis owing to the limited sampling timepoints. Mean pharmacokinetic parameters for arterolane maleate and piperaquine phosphate following three-day oral administration from intensive sampling are presented in Table 6. The mean t_1/2_ values for arterolane maleate and piperaquine phosphate were 3.3 and 292.72 h, respectively. Pharmacokinetic results through non-compartmental analysis indicated that mean exposures of arterolane maleate and piperaquine phosphate in children were similar across three different age groups in this study. Accumulation for arterolane maleate was not observed following three-dose treatment regimes, however, accumulation for piperaquine phosphate was observed based on its longer terminal t_1/2_. A relationship of PCT as a function of C_max_ of arterolane maleate was evaluated as depicted in Fig. 5. The distribution of PCT appeared to be fairly uniform which indicated that, among the patients evaluated, there is no potential relationship of PCT with C_max_ of arterolane maleate. Additionally, PCT as a function of arterolane maleate exposure (AUC_last_) was evaluated as shown in Fig. 6 and it appeared that arterolane maleate exposure levels (AUC_last_) of less than ~5 µg h/mL were potentially sufficient to result in a PCT ranging from six to 36 h. In this study, 48 patients (n = 58) on day 7 had mean concentration for piperaquine phosphate greater than 30 ng/mL.

**Table 6:** Mean pharmacokinetic parameters following 3 day oral administration of arterolane maleate and piperaquine phosphate

Analyte	Parameters	C_max_ (ng/mL)	AUC_last_ (ng h/mL)	AUC_48-72_ (ng h/mL)	t_1/2_ (h)
	n	50	50	48	26
Arterolane maleate	AM	90.68	1507.93	688.29	3.30
	Median	83.65	1209.30	615.25	3.24
	Minimum	23.38	110.74	137.71	2.04
	Maximum	204.96	6174.14	1696.75	5.48

	n	50	50	48	48
Piperaquine phosphate	AM	578.97	58,472.41	7333.04	292.72
	Median	497.77	50,453.06	6417.42	221.48
	Minimum	58.71	965.76	2337.00	94.60
	Maximum	1604.18	169,956.99	20,590.96	801.86

*AM* arithmetic mean

### Safety evaluation

The most frequent clinical adverse event was vomiting reported in 35 patients (24.8 %) (Table 7). Most of the adverse events (94 %) were of mild to moderate in intensity. There were no deaths and serious adverse events reported during the course of the study.

**Table 7:** Adverse events

	Age group 1 (6 months to <2 years)	Age group 2 (2 to <6 years)	Age group 3 (6 to ≤ 12years)	Total (n = 141)
No. of patients with at least one adverse event	22 (100 %)	49 (100 %)	68 (97.1 %)	139 (98.6 %)
Clinical adverse events				
Vomiting	9 (40.9 %)	21 (42.9 %)	5 (7.1 %)	35 (24.8 %)
Anaemia	1 (4.5 %)	7 (14.3 %)	13 (18.6 %)	21 (14.9 %)
Cough	2 (9.1 %)	4 (8.2 %)	2 (2.9 %)	8 (5.7 %)
Pyrexia	0 (0.0 %)	2 (4.1 %)	2 (2.9 %)	4 (2.8 %)
Diarrhoea	1 (4.5 %)	1 (2.0 %)	0 (0.0 %)	2 (1.4 %)
Otitis media	1 (4.5 %)	0 (0.0 %)	1 (1.4 %)	2 (1.4 %)
Headache	0 (0.0 %)	0 (0.0 %)	2 (2.9 %)	2 (1.4 %)
Hyperhidrosis	0 (0.0 %)	2 (4.1 %)	0 (0.0 %)	2 (1.4 %)
Abdominal pain	0 (0.0 %)	1 (2.0 %)	0 (0.0 %)	1 (0.7 %)
Nausea	0 (0.0 %)	1 (2.0 %)	0 (0.0 %)	1 (0.7 %)
Asthenia	0 (0.0 %)	1 (2.0 %)	0 (0.0 %)	1 (0.7 %)
Rhinitis	0 (0.0 %)	1 (2.0 %)	0 (0.0 %)	1 (0.7 %)
Varicella	0 (0.0 %)	1 (2.0 %)	0 (0.0 %)	1 (0.7 %)
Productive cough	1 (4.5 %)	0 (0.0 %)	0 (0.0 %)	1 (0.7 %)
Decreased appetite	0 (0.0 %)	1 (2.0 %)	0 (0.0 %)	1 (0.7 %)

The mean increase in QTc interval over baseline on day 1 and day 2 were 20.3 and 21.3 ms, respectively. None of patients had QTc >500 ms on days 1 and 2. QTc prolongation was noted in 16 patients. Of these, 12 (8.5 %) patients had QTc >60 ms increase over baseline on day 1 while ten (7.1 %) patients had QTc >60 ms increase over baseline on day 2. The increase in mean QTc from baseline was not clinically significant as judged by investigator. None of the patients with prolonged QTc (>60 ms from baseline) had associated *torsade de pointes* or polymorphic ventricular tachycardia or signs/symptoms of serious arrhythmia and there were no withdrawals from the study based on QTc prolongation. A total of 6 events of QTc prolongation were judged as severe by the investigator and these events resolved without sequelae or improved by day 14.

Haematological results were analysed for all patients (Table 8). There was a decrease in mean haemoglobin and haematocrit levels during the first 72 h of treatment with complete recovery to initial levels by day 42. In two patients, there was a clinically significant decrease in haemoglobin during the first 48 h, which subsequently increased and exceeded the baseline value by day 42. A rise in platelet, eosinophil, lymphocyte, monocyte counts, and downward trend in white blood cells, neutrophils was noted during the study. There were no significant changes in the biochemical parameters during the course of the study other than one event of severe hyperkalaemia which started on day 28 and resolved without sequelae by day 42.

**Table 8:** Haematological parameters (mean ± SD)

Parameter (unit)	Day 0	Day 2	Day 7	Day 28	Day 42
Mean haemoglobin (g/dL)	10.8 ± 1.49	9.7 ± 1.36	10.3 ± 1.39	11.4 ± 1.00	12.2 ± 1.47
Mean haematocrit	32.1 ± 4.27	28.9 ± 4.61	31.0 ± 4.19	34.5 ± 3.19	35.9 ± 3.98
Platelet count (1000/mm^3^)	151.5 ± 77.26	175.3 ± 65.65	325.4 ± 126.44	268.6 ± 83.53	311.9 ± 104.77
Lymphocyte (%)	29.5 ± 13.38	46.9 ± 11.88	44.8 ± 11.83	44.3 ± 11.95	45.3 ± 12.48
Eosinophils (%)	1.9 ± 2.39	3.2 ± 4.17	4.3 ± 3.96	5.8 ± 6.18	5.5 ± 5.54
Monocytes (%)	6.7 ± 5.64	8.4 ± 6.15	8.6 ± 4.55	7.5 ± 4.80	7.9 ± 5.50
White blood cells (1000/µL)	8.1 ± 3.08	7.3 ± 2.41	7.9 ± 2.92	7.2 ± 2.06	7.2 ± 2.23
Neutrophils (%)	61.7 ± 15.58	41.2 ± 14.70	42.2 ± 12.91	42.2 ± 13.84	41.2 ± 13.91

## Discussion

In response to increased resistance of malaria parasites to conventional drugs, WHO recommends ACT for uncomplicated malaria treatment [2]. A new FDC of arterolane maleate 150 mg and piperaquine phosphate 750 mg tablets has been approved in India for the treatment of falciparum and vivax malaria in adults. A FDC of arterolane maleate 37.5 mg and piperaquine phosphate 187.5 mg dispersible tablets has been formulated and this study evaluated the efficacy and safety in paediatric patients aged 6 months to 12 years having falciparum malaria.

The PCR-corrected ACPR on day 28 was achieved in 100 % patients in per-protocol population in this study and the results accord with the high activity reported for other ACT in children with falciparum malaria [11, 14–21]. The efficacy is consistent with the phase III study of FDC of arterolane maleate and piperaquine phosphate tablets conducted in patients of falciparum malaria aged 12–65 years (PCR-corrected ACPR rates at day 28: 99.25 %). There were no treatment failures until day 28 in all the three age groups in this study.

It was recommended by WHO that the proportion of patients with parasite clearance on day 3 currently as the best available indicator used in routine monitoring to measure *Plasmodium falciparum* sensitivity to artemisinins and the efficacy of ACT [22]. The artemisinin component in the FDC, arterolane maleate has been reported to achieve rapid parasite clearance within 3 days [5, 23] which is further validated by median PCT of 24.0 h and the median FCT of 10 h in this study.

The concentrations of slowly eliminated anti-malarial drugs are measured routinely on day 7 because this reflects the degree of drug exposure that must kill residual malaria parasites for an effective cure. Low piperaquine phosphate concentrations on day 7 are predictive of therapeutic failure. It has been hypothesized that a concentration greater than 30 ng/mL on day 7 for piperaquine phosphate could be an indicator of low probability of recrudescence [24, 25]. In this study, the majority of patients on day 7 had mean concentration for piperaquine phosphate greater than 30 ng/mL. This indicates that concentrations of piperaquine phosphate following this dosage regimen in children could be potentially sufficient to prevent recrudescence. This is further substantiated by the fact that no recrudescence was reported at day 28 in the per protocol population in this study.

The piperaquine phosphate pharamacokinetic parameters well corroborated with the values reported in literature [25–27].

In the current study, a high incidence of vomiting (24.8 %) was reported and is consistent with incidence rates reported in the literature on paediatric patients taking anti-malarial drugs [16, 18, 19]. An incidence of 20.2 % for vomiting was reported with FDC of artemether-lumefantrine in infants and children of 12 years of age and below [28]. Similarly, anaemia rates (14.9 %) reported in this study are consistent with rates (up to 17.2 %) reported with other ACT in children [18, 20].

In this study, none of patients had QTc >500 ms. Increase in mean QTc >60 ms from baseline was reported in few patients, which were not associated with *Torsade de pointes* or polymorphic ventricular tachycardia or signs/symptoms of serious arrhythmia and there were no withdrawals from the study based on QTc prolongation. Evaluation of QT interval changes is problematic in malaria because of systematic differences between the acute febrile admission before anti-malarial drugs are given and early convalescence. At presentation, patients are usually anxious, fasting and febrile with increased autonomic tone and a raised heart rate. This contrasts with the relaxed fed, supine, afebrile state 3 days later when most anti-malarial treatments are completed and anti-malarial concentrations are at the highest. It has been argued that this systematic reduction in sympathetic activity with recovery leads to a consistent increase in the QT interval, which has been mistakenly ascribed to anti-malarial drug effects. It has been reported in the literature that QTc prolongation of >60 ms was within the bounds of normal daily variation in the QTc (up to 75–100 ms) [29, 30].

Most adverse events were mild to moderate in intensity, with gastrointestinal symptoms being most frequently reported clinical adverse events. There were no deaths and serious adverse events reported during the course of the study. The safety profile in this study well corroborated with that reported in published literature in patients with malaria [31–35]. Despite the relatively modest number of patients that this study considered, it is reassuring that no safety concerns occurred during the study.

## Conclusion

The findings from this study showed that FDC of arterolane maleate and piperaquine phosphate effectively cures falciparum malaria and attains acceptable level of cure by day 28 in paediatric patients. The efficacy and safety results observed in children warrants further studies with larger population on FDC of arterolane maleate and piperaquine phosphate dispersible tablets.

## Abbreviations

ACPR: Adequate clinical and parasitological response
ACT: Artemisinin-based combination therapy
AM: Arterolane maleate
ECG: Electrocardiogram
ETF: Early treatment failure
FCT: Fever clearance time
FDC: Fixed dose combination
ITT: Intent to treat
LCF: Late clinical failure
LC–MS/MS: Liquid chromatography coupled with tandem mass spectrometry mass spectrometry
LPF: Late parasitological failure
PQP: Piperaquine phosphate
PCR: Polymerase chain reaction
PCT: Parasite clearance time
QTc: Corrected QT interval
ULN: Upper limit of normal
WHO: World Health Organization
